# Pharmacokinetics of Acetyl-L-Carnitine Given in Single or Multiple Doses to HIV-1 Infected Patients with Toxic Peripheral Polyneuropathy

**DOI:** 10.2174/1874613600802010039

**Published:** 2008-06-04

**Authors:** C Herzmann, S.J Whiting, M Thomas, P. Byrne, M.A Johnson, M Youle

**Affiliations:** 1 Vivantes Auguste Viktoria Klinikum, Rubensstr. 125, D-12157 Berlin, Germany; 2 Royal Free Hospital, Centre for HIV Medicine, Royal Free Hospital, Pond Street, London, NW3 2QG, UK

## Abstract

The use of nucleoside reverse transcriptase inhibitors in the treatment of HIV infection is associated with antiretroviral toxic polyneuropathy (ATN). Previous studies suggest that long term treatment with Acetyl-L-carnitine (ALCAR) 1.5 gram twice daily improves symptoms and promotes nerve regeneration. It is unknown whether the drug’s pharmacokinetic profile would allow for a once daily administration. Twenty three HIV-1 infected subjects taking ALCAR for ATN were enrolled in a cross over trial and switched from twice to once daily dosing. Their regimen was changed from 1.5g twice daily to 1g (4 patients), 2g (7), and 3g (12) once daily, respectively. Twelve healthy volunteers served as control. Plasma levels of ALCAR and its metabolite L-carnitine were measured. Patients receiving ALCAR had higher pre-dose levels than control subjects. Post dose levels were not significantly higher than pre dose levels in any treatment group. The pre / post dose ALCAR concentrations were 7.6 / 7.7, 7.1 / 6.8, 7.7 / 6.8, and 7.1 / 7.5 µmol/l for 1.5g twice daily, 1g once daily, 2g once daily, and 3g once daily, respectively. All values were significantly higher than the mean concentration in the control group (4.3 µmol/l). For ALCAR and L-carnitine, measurements for once daily regimens did not differ from the twice daily regimen. Once daily dosing of ALCAR can achieve similar plasma levels as twice daily dosing but intra-mitochondrial levels remain unknown. The pharmacokinetic profile of orally administered ALCAR is complex and likely to be highly affected by endogenous concentrations.

## INTRODUCTION

Highly active antiretroviral therapy (HAART) is the current standard treatment for HIV infection but is associated with long term side effects. Nucleoside reverse transcriptase inhibitors (NRTI’s), a major component of HAART, are associated with distal symmetrical polyneuropathy (DSP) [1]. This antiretroviral toxic neuropathy (ATN) occurs mainly but not exclusively with dideoxynucleotide analogue agents, e.g. stavudine and didanosine, and causes significant morbidity in 10-35% of patients [2,3].

NRTI’s interfere with mitochondrial metabolism by reducing neuronal mitochondrial DNA synthesis leading to a disruption of oxidative meta-bolism [1,4-6]. Subsequently, peripheral axons die back as the neuronal mitochondria are unable to meet their metabolic requirements. Histological examination confirms reduced epidermal innervation in affected areas [7,8].

Acetyl-L-carnitine (ALCAR) is an ester of L-carnitine and plays a major role in normal mitochondrial function, being a transport molecule for free fatty acids and an important acetyl-group donor in high-energy metabolism and free fatty acid beta-oxidation [9,10]. Its main body stores are in skeletal and cardiac muscle. It is found along with free plasma L-carnitine and other acyl-esters of varying chain length [11]. The formation of ALCAR originates with cytoplasmic thiokinase which forms acyl-Coenzyme A from free fatty acids, ATP and Coenzyme A (CoA) [12]. This substance is combined with carnitine to form acetyl-carnitine *via *carnitine palmitoyltransferase I. Entry into the mitochondrial matrix occurs through an exchange system of acylcarnitine / carnitine *via *carnitine-acylcarnitine translocase. For each acylcarnitine molecule traversing the inner mitochondrial membrane, a molecule of carnitine is shuttles out. On the inner mitochondrial membrane, carnitine palmitoyltransferase II converts acylcarnitine to carnitine, liberating acylCoA. Finally, the production of ALCAR and CoA from carnitine and acetylCoA (obtained *via *beta-oxidation of acylCoA) occurs *via *carnitine acetyltransferase present in the mitochondrial matrix [12,13]. The enzymatic formation of ALCAR in the mitochondrial matrix is reversible, releasing free Coenzyme A and acetylCoA which can readily be exchanged across membranes, thus providing metabolic energy to intracellular organelles.

ALCAR plasma levels are decreased in HIV positive patients suffering from ATN [14]. It appears that certain pathways of carnitine metabolism, such as the acetylation of carnitine to acetyl-carnitine, are impaired, possibly as a result of the toxicity of antiretroviral medication.

Oral treatment with ALCAR (1.5g twice daily) has shown some beneficial effect on symptoms and nerve regeneration in patients with ATN [15-18]. It is unknown whether the pharmacokinetic profile would allow a once daily administration.

## METHODS

The study was approved by the Royal Free Ethics Committee, London. Consent was obtained from all patients. The inclusion criteria for this cross over trial were: Male or female patients with confirmed HIV-1 infection (ELISA antibody testing), a stable antiretroviral combination that remained unchanged throughout the study, a clinical diagnosis of antiretroviral toxic neuropathy, and ALCAR therapy (1.5 g twice daily) for at least 3 months. Plasma samples for the measurement of ALCAR and L-carnitine levels were taken pre-dose (fasting) and 2-hour post-dose. Subsequently, the twice daily dose was changed to a range of once daily doses (1g, 2g, and 3g). Plasma sampling was repeated after 4 weeks.

Healthy volunteers, recruited from laboratory staff served as control. None of them was known to be HIV infected nor taking ALCAR.

Sensitive high pressure liquid chromatography (HPLC) with fluorometric detection as described elsewhere [19], was used for the measurement of ALCAR and L-carnitine in blood plasma. The procedure involves pre-column derivitisation with 1-aminoanthrecene performed in phosphate buffer in the presence of 1-(3-dimethylaminopropyl)-3-ethylcar-bodiimide hydrochloride as catalyst. The fluorescent derivatives were isocratically separated on a reversed-phase column. The eluate was monitored with fluorometric detector set at 248 nm (excitation wavelength) and 418 nm (emission wavelength). At the concentrations measured, the assay has been found to have an imprecision of less than 10%, as determined in preceding measurements. All plasma samples were processed and analysed in duplicate. A standard curve was plotted prior to each analytical run.

The data was normally distributed. A Student’s t-test was used for analysis with statistical significance set at p<0.05.

## RESULTS

Twenty-three HIV infected subjects on ALCAR therapy and 12 HIV uninfected control subjects were enrolled and completed the study. There were slightly more men in the treatment group and the control group was younger (Table **1**). Six patients in the treatment group were taking one NRTI, 13 were taking two NRTIs, 3 were taking 3 NRTIs in combination with 1-2 protease inhibitors (PI) and / or non-nucleoside reverse transcriptase inhibitors (NNRTI). One patient was taking an antiretroviral regime consisting of a PI and an NNRTI only. From 1.5 g ALCAR twice daily (23 patients, plasma levels from 15 patients obtained), the treatment was changed to once daily doses of 1g (4 patients), 2g (7 patients), or 3g (12 patients), respectively.

The plasma levels are shown in Table **2** and Figs. (**1**) and (**2**). When compared with the control group, all ALCAR plasma levels (pre and post dose) were significantly higher in the treatment groups (p < 0.02). For L-carnitine, all post dose level were significantly higher than in the control group (p < 0.03). Pre dose levels, however, were only significantly higher in the groups taking 1.5g twice daily (p = 0.03) or 2g once daily (p = 0.008). Accordingly, post dose levels were significantly higher than pre dose levels in patients taking 1g or 3g once daily (p <0.05). There was no significant difference between any of the plasma levels (ALCAR and L-carnitine) in the treatment groups. In particular, there was no difference between once daily and twice daily regimen.

## DISCUSSION

As previously shown, plasma ALCAR levels are decreased in patients with ATN [14,20]. Oral ALCAR supplementation seems to result in plasma levels that are higher than in healthy individuals. Our findings may therefore provide indirect support for reports that ALCAR promotes nerve fibre regeneration and can improve neuropathic symptoms [15-18].

No significant differences in plasma levels were seen in the different dosage groups despite a reduction of the total daily dose by up to two thirds (from 1.5g twice daily to 1g once daily). However, our study size was small and its results need to be interpreted with caution. The metabolism of ALCAR and L-carnitine is complex. It has been shown that peripheral blood monocytes in AIDS patients are low in intracellular L-carnitine [21]. Serum levels, however, may be high, low or normal and are therefore unreliable as an indicator of L-carnitine metabolism [22].

L-carnitine and ALCAR homeostasis are maintained by absorption from diet, a modest rate of synthesis, very efficient renal re-absorption and probably renal tubular secretion. Dietary ALCAR is hydrolysed partially during absorption in enterocytes, partially after absorption. Both ALCAR and L-carnitine plasma concentrations have been found to return to baseline within 12 hours after injection [23]. The key mechanism for this seems to be renal re-absorption that displays saturation kinetics. With rising L-carnitine concentrations, re-absorption decreases and clearance increases. This results in a rapid decline of circulating L-carnitine. Elimination kinetics for ALCAR are similar to those for L-carnitine [23].

Our data suggest that oral supplementation of ALCAR may increase the steady state concentration of ALCAR in blood plasma until its saturated renal kinetics prevent a further rise. However, continuous treatment with ALCAR may lead to mitochondrial storage which could explain why there is no significant difference between pre and post dose levels in most treatment groups. It may also explain why plasma levels remained elevated for more than 12 hours in contrast to reports on intravenous application [23].

However, it remains unknown whether high plasma levels are associated with high intra-mitochondrial levels. Given that the pathogenesis of antiretroviral toxic neuropathy is based on the disruption of mitochondrial energy supply one can assume that the clinical effect of ALCAR is dependent on its mitochondrial availability. There are, unfortunately, no readily available tests to evaluate mitochondrial toxicity of NRTIs nor intra-mitochondrial concentrations of ALCAR.

It appears that once daily and twice daily doses of ALCAR can achieve similar plasma levels. Therefore the easier administration schedule may be equally effective for the treatment of antiretroviral toxic neuropathy. Trials are needed to compare the clinical effect on symptoms and nerve regeneration.

## Figures and Tables

**Figure:**
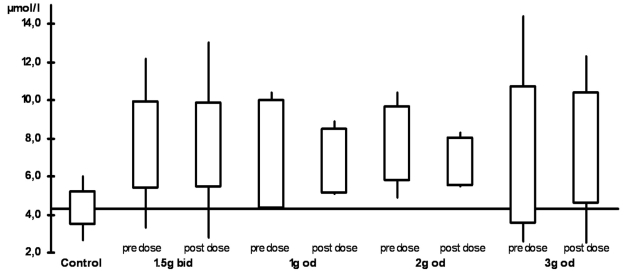
ALCAR plasma levels (bid: twice daily,od: once daily)

**Fig.(2):**
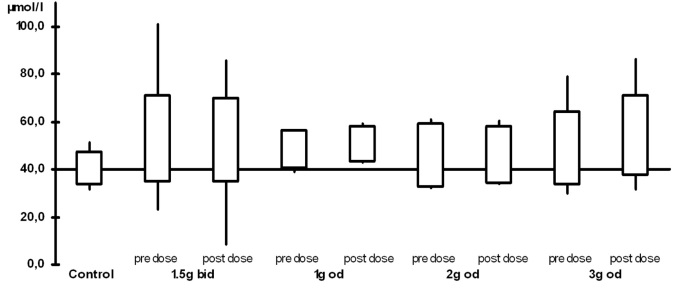
L-carnitine plasma levels (bid: twice daily, od: once daily)

**Table 1:** Baseline Characteristics (SD: Standard Deviation)

	ALCAR Group (n=23)	Control Group (n=12)
Men ;#x0025;	87	75
Mean age in years (range)	43 (34 to 60)	35 (27 to 62)
Time of HIV infection in years (SD)	10.5 (5.1)	NA
Subjects with previous HIV related disease	7	0
Patients with <50 cc/ml viral load	12	12

**Table 2:** Mean Plasma Levels in µ5mol/l (Standard Deviation)

		1.5g ALCAR Twice Daily	1g ALCAR Once Daily	2g ALCAR Once Daily	3g ALCAR Once Daily	Control Group
		(n=15*)	(n=4)	(n=7)	(n=12)	(n=12)
ALCAR	pre-dose	7.6 (2.3)	7.1 (2.9)	7.7 (2.0)	7.1 (3.6)	4.3 (0.9)
	2hrs post-dose	7.7 (2.2)	6.8 (1.7)	6.8 (1.3)	7.5 (2.9)	
L-carnitine	pre-dose	52.8 (18.3)	48.3 (8.1)	51.0 (8.2)	49.0 (15.5)	40.2 (7.0)
	2hrs post-dose	52.1 (17.9)	50.6 (7.5)	53.0 (5.0)	54.3 (16.9)	

^*^ Values for 15 of 23 patients were obtained.

## ABBREVIATIONS

ALCAR: Acetyl-L-Carnitine
ATN: Antiretroviral toxic neuropathy
bid: Twice daily
CoA: Coenzyme A
DSP: Distal symmetrical polyneuropathy
ELISA: Enzyme linked immunosorbent assay
NRTI: Nucleoside reverse transcriptase inhibitors
od: Once daily
